# PET assessment of acute gastrointestinal graft *versus* host disease

**DOI:** 10.1038/s41409-023-02038-9

**Published:** 2023-08-03

**Authors:** Ashleigh P. Scott, Andrea Henden, Glen A. Kennedy, Siok-Keen Tey

**Affiliations:** 1 Department of Haematology and Bone Marrow Transplant, Royal Brisbane and Women’s Hospital, Brisbane, QLD Australia; 2 School of Medicine, The University of Queensland, Brisbane, QLD Australia; 3 QIMR Berghofer Medical Research Institute, Brisbane, QLD Australia

**Keywords:** Graft-versus-host disease, Medical imaging

## Abstract

Acute gastrointestinal graft *versus* host disease (GI-GVHD) is a common complication following allogeneic haematopoietic cell transplantation (HCT), and is characterised by severe morbidity, frequent treatment-refractoriness, and high mortality. Early, accurate identification of GI-GVHD could allow for therapeutic interventions to ameliorate its severity, improve response rates and survival; however, standard endoscopic biopsy is inadequately informative in terms of diagnostic sensitivity or outcome prediction. In an era where rapid technological and laboratory advances have dramatically expanded our understanding of GI-GVHD biology and potential therapeutic targets, there is substantial scope for novel investigations that can precisely guide GI-GVHD management. In particular, the combination of tissue-based biomarker assessment (plasma cytokines, faecal microbiome) and molecular imaging by positron emission tomography (PET) offers the potential for non-invasive, real-time in vivo assessment of donor:recipient immune activity within the GI tract for GI-GVHD prediction or diagnosis. In this article, we review the evidence regarding GI-GVHD diagnosis, and examine the potential roles and translational opportunities posed by these novel diagnostic tools, with a focus on the evolving role of PET.

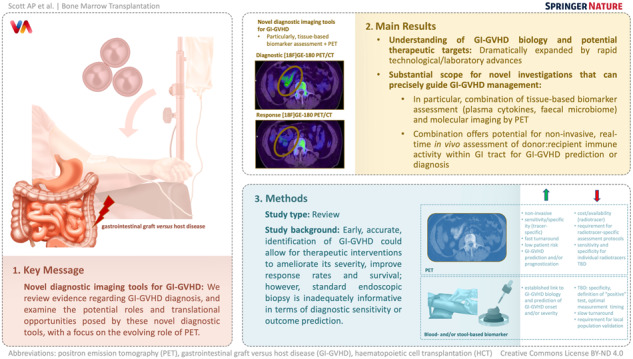

## Gastrointestinal acute graft *versus* host disease (GI-GVHD)

Despite advances in donor selection and prophylaxis strategies, acute graft *versus *host disease (aGVHD) [1–4] still occurs in around 40% (30–80%) of allogeneic haematopoietic cell transplantation (HCT) recipients [5–8]. The pathophysiology of aGVHD involves conditioning-associated tissue damage, release of proinflammatory cytokine by recipient tissues, activation of donor immune cells, and consequent immune-mediated destruction of recipient target tissue, primarily the skin, liver, and gastrointestinal (GI) tract [4, 9–13]. GI-GVHD [3, 4] accounts for up to 40% of aGVHD [3, 5–8, 11, 14, 15], and is clinically characterised by the rapid onset of profuse secretory diarrhoea, frequently resulting in death from haemorrhage, perforation, or secondary infection [11, 16, 17]. Clinical presentation, severity and prognosis differ depending upon GI tract regions involved [18]. GI-GVHD is a clinical diagnosis based upon consideration of patient risk factors (Table 1) and exclusion of differential diagnoses, such as conditioning-associated mucositis, infections and cytomegalovirus (CMV) colitis [4, 16].

**Table 1:** Risk factors for aGVHD.

Risk factor	Subgroup at increased GVHD risk	Reference
Age	Older recipients	[6, 79]
Donor other than HLA-matched sibling	Matched unrelated, mismatched and/or haploidentical donors	[6, 8, 80, 81]
HPC source	Peripheral blood stem cells	[5]
Conditioning	Myeloablative	[8]
Sex mismatch	Female donor to male recipient	[6]
Donor gravidity	Recipients from multiparous donors	[6, 82–85]
Intolerance of immune suppression	Early organ failure, e.g. renal/hepatic, necessitating unplanned modifications to GVHD prophylaxis	[86]
GI tract microbiome	Decreased diversity post-HCT associated with increased incidence of GI-GVHD	[74–76]

*HPC* haematopoietic progenitor cells, *HLA* human leucocyte antigen, *GI* gastrointestinal.

## Endoscopic biopsy

Endoscopic examination and biopsy is used to confirm or refute GI-GVHD [16]. Macroscopic endoscopic findings include oedema, erythema, erosion, ulceration, and sloughing [16, 19, 20]; however, macroscopically normal mucosa frequently contains histological GI-GVHD and should also be biopsied [16, 19, 21, 22]. Histologically, the characteristic finding is apoptosis of mucosal epithelium [23–25], with sloughing and denudation in severe cases [25–28].

Whilst endoscopic biopsy is the accepted “gold standard” for GI-GVHD diagnosis, numerous limitations negatively impact upon its diagnostic sensitivity and specificity, resulting in up to 26% of patients requiring treatment for GI-GVHD despite negative endoscopic biopsies [29]. Pre-test probability of endoscopically identifying GI-GVHD is influenced by institutional practices regarding GVHD prophylaxis [5–8] and extent of endoscopy procedures [21, 22, 29–33]. Sampling error is compounded by heterogeneous GI tract involvement by GI-GVHD, where involved sites may be macroscopically normal [21, 30, 31] or inaccessible [16]. Notably, however, sensitivity of upper endoscopy for GI-GVHD appears relatively low [22, 29, 32–34] and routine omission of this procedure results in few missed cases of isolated upper GI-GVHD [29, 35]. Histologic results can be non-specific [4, 16], correlate poorly with clinical severity [29], and can take 48–72 hours to determine [16, 21, 22, 28], thus potentially delaying treatment.

Novel endoscopic techniques have been examined to improve diagnostic sensitivity. Wireless capsule endoscopy (WCE) identifies small bowel  macroscopic GI-GVHD lesions in concordance with distal lesions identified during conventional endoscopy; addition of confocal laser endomicroscopy (CLE) can also detect sub-macroscopic changes [36, 37]. Endoscopic ultrasound (EUS) with double balloon enteroscopy (DBE) allows visualisation of the small bowel [38]. Fundamentally, however, these procedures do not overcome the essential limitations of endoscopy itself: the GI tract is not fully available for endoscopic biopsy, and thus histologic confirmation of GI-GVHD is subject to sampling error [17].

## Conventional GI tract imaging

The potential advantage of GI tract imaging over endoscopy is the capacity to non-invasively assess the entire GI tract, including endoscopically inaccessible regions. Indeed, if an imaging biomarker was sufficiently accurate, it could potentially diagnose GI-GVHD without histologic biopsy. The challenge, however, has been to develop imaging modalities with sufficient specificity for GI-GVHD.

Conventional imaging techniques such as computed tomography (CT) or magnetic resonance imaging (MRI) are limited by their inability to detect microscopic or subtle macroscopic features of GI-GVHD. Imaging features of GI-GVHD are similar to those seen in any inflammatory or infectious enteritis: these include bowel wall thickening, mural stratification, mucosal enhancement, oedema, and mesenteric stranding [39, 40]. These have relatively poor sensitivity and specificity for GI-GVHD in clinical studies. Plain CT or MRI can support a clinical or histological diagnosis of GI-GVHD, predict its onset, and identify radiologically severe features, but does not improve diagnostic sensitivity or aid prognostication beyond existing clinical and endoscopic methods [41–45]. Attempts to improve sensitivity for subtle lesions and describe functional impact by mapping the luminal GI tract with oral contrast (enterography) have been limited by poor patient tolerance and inability to differentiate between GI-GVHD and other causes of colitis [39]. MRI provides greater resolution than CT but is limited by a relatively long scan duration (30–60 min) and the requirement for all metal (such as infusion pumps for antibiotics, analgesia, or parenteral nutrition) to be disconnected from the patient. Currently, CT/MRI+/− enterography is not routinely employed for GI-GVHD diagnosis in most centres and is not considered to be a requirement for GI-GVHD diagnosis.

Ultrasound is an attractive imaging modality because it is non-invasive, radiation-free, relatively inexpensive, and can be performed by the bedside. Ultrasound can be used to assess GI tract wall thickness, dilatation, and obstruction, and also has the advantage of highlighting the functional impact of GI-GVHD lesions. Retrospective, non-randomised studies evaluating conventional US performed contemporaneously with diagnostic endoscopy reported high sensitivity but relatively lower specificity, with up to one quarter false positives, without any clear single sonographic feature that could reliably differentiate GI-GVHD from mucositis or CMV colitis [46]. Modified ultrasound could improve specificity: contrast-enhanced ultrasound (CEUS) allows mapping of GI tract microvasculature and perfusion following intravenous injection of contrast microbubbles [47, 48], and compound elastography (CE) assesses luminal stiffness as a measure of inflammation. In a prospective study in patients with biopsy-proven GI-GVHD compared to asymptomatic HCT recipient controls, CEUS and CE had a 92.9% sensitivity and 94.4% specificity for GI-GVHD [49]. Notably, however, this study’s pilot design did not allow CEUS to differentiate GI-GVHD from other differential diagnoses (such as CMV colitis) in symptomatic patients, and thus the reported specificity for GVHD in a clinical context needs confirmation in larger studies. Interestingly, diagnostic sensitivity was not improved by the additional measurement of serum biomarkers such as REG3α. CEUS has been used to monitor GI-GVHD response to therapy with only modest correlation [50].

Overall, the principal limitation of conventional imaging techniques for GI-GVHD is that they only identify macroscopic anatomic changes, which are often similar between GI-GVHD and differential diagnoses. Thus, conventional imaging provides essentially similar information as endoscopy, albeit derived from the entire GI tract. Novel imaging techniques such as positron emission tomography (PET), however, can take advantage of cellular and molecular changes in GI-GVHD.

## Positron emission tomography (PET)

Molecular imaging using PET [51] could potentially improve the specificity of conventional imaging by visually identifying tissue GI-GVHD biomarkers. A PET radiotracer consists of a radioisotope that is chemically bound to a ligand of the molecular biomarker. Following intravenous administration, the radiotracer binds to any target molecule accessible to the patient’s bloodstream. The avidity of radiotracer uptake is visualised on PET, mathematically calculated, and reported as the maximum standardised uptake variable (SUVmax). Anatomic localisation is assisted by simultaneous low-dose CT (PET/CT) or MRI (PET/MRI) for visual correlation. PET images are interpreted by qualitative (visual) and quantitative (SUV) assessment of avidity in suspected pathological tissues, and compared to background physiologic avidity in a non-pathological “reference” tissue, such as the mediastinal blood pool or liver. PET can precisely differentiate normal and abnormal tissues even within organs that may otherwise appear macroscopically normal if the target molecule is significantly and specifically increased in the pathology of interest. PET is thus attractive for diagnosing GI-GVHD because of its potential to non-invasively demonstrate molecular evidence of GI-GVHD across the entire length of the GI tract, particularly when histologic or macroscopic evidence is otherwise lacking or inaccessible.

The utility of PET is dependent upon the target molecule and selected radiotracer. Most PET scans employ [^18^F]fluorodeoxyglucose ([^18^F]FDG), a glucose-targeting radiotracer that is taken up by any metabolically active cell. As glucose uptake is generally increased in active malignancies, infections and inflammation, [^18^F]FDG PET/CT is in routine use as a staging modality for many malignancies [51–53]. However, whilst [^18^F]FDG is frequently manufactured and thus reasonably inexpensive and ubiquitous, it is limited by its lack of biological specificity for individual diseases; anatomic regions containing increased avidity need to be interpreted in the appropriate clinical or histological context.

### [^18^F]FDG PET in GI-GVHD

Until recently, most PET research in GI-GVHD has been conducted using [^18^F]FDG. Prospective clinical trials of diagnostic [^18^F]FDG PET/CT, performed within 7 days of endoscopy and biopsy for clinically suspected GI-GVHD, yielded sensitivity and specificity similar to those of endoscopic biopsy (71% and 82% respectively) [54, 55], however with a low PPV (57%). The most comprehensive study, reported recently by Cherk et al. [56], prospectively enroled 51 patients with newly suspected GI-GVHD; subjects underwent 4 upper and 4 lower GI tract biopsies and an [^18^F]FDG PET/CT prior to commencing corticosteroid therapy. Sensitivity was ~70%, and specificity 57% and 76% for quantitative and qualitative assessment respectively, with non-GVHD inflammation demonstrating consistently higher SUVmax than GI-GVHD. Surveillance [^18^F]FDG PET/CT, performed routinely at the time of neutrophil recovery following HCT, can aid prediction of subsequent clinical GI-GVHD [57, 58], with similar potential pitfalls as per the surveillance approach used in cytokine biomarker studies. Subsequent retrospective analyses report high sensitivity (93%) but relatively poorer specificity and negative predictive value (73% and 64% respectively) [59]. Although [^18^F]FDG shows promising clinical potential and is relatively inexpensive compared to other radiotracers, its lack of specificity as a single modality is the major limitation.

### Novel radiotracers in GI-GVHD

An inherent challenge in developing novel PET radioligands in GI-GVHD is that there is no one “specific” molecular hallmark. Nevertheless, PET using radiotracers that identify immunological components of the activation and effector phases of aGVHD, such as donor T-cells, are of great interest. Early attempts at PET radiotracer development in GI-GVHD targeted markers of activated T-cells such as HLA-DR [60], CD3 [61] and FLT3 [62]; these studies illustrated proof of concept but were limited by lack of specificity.

Recent studies have reported more promising results. [^18^F]AraG is a compound that is taken up by activated T-cells, and is cytotoxic when given at therapeutic doses. In a mouse model of aGVHD, [^18^F]AraG PET/CT identified activated T-cells in secondary lymphoid organs prior to aGVHD onset [63]. The potential advantage of this radiotracer would be identification of high-risk patients prior to aGVHD onset, rather than at the time of clinical symptoms, thus potentially enabling early intervention. This radiotracer is currently being evaluated in a clinical trial (NCT03367962). The same group recently reported the use of a novel radiotracer comprising a monoclonal antibody against OX40 (CD134), a T-cell costimulatory molecule that is a marker of activated T-cells [64]. Using a similar mouse model and study design, the authors demonstrated that [^64^Cu]OX40mAb PET/CT consistently identified OX40+ activated T-cells in lymphoid organs and the GI tract prior to aGVHD development. However, the antibody clone used in [^64^Cu]OX40mAb radiotracer appears unsuitable for clinical application because it was agonistic and thus increased aGVHD onset time and lethality. Antagonist or non-agonist OX40 monoclonal antibody clones will be required for any future studies.

**Fig. 1:**
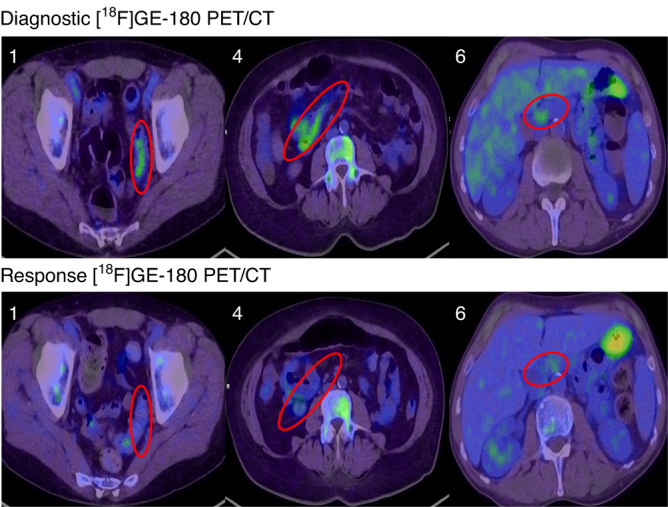
Examples of positive PET using [^18^F]GE-180. Representative “True Positive” axial images of [^18^F]GE-180 PET/CT, reproduced with permission from Scott et al. (full details available at 10.1038/s41409-022-01571-3) [66]. Diagnostic imaging was performed at GI-GVHD diagnosis and repeated 7-14 days following treatment commencement, to evaluate correlation with diagnostic histology and clinical response to corticosteroid treatment respectively. Top panel contains study participants’ (1, 4 and 6) “Diagnostic” images, bottom panel contains corresponding “Response” images. Diagnostic [^18^F]GE-180 PET/CT: increased activity in sigmoid colon (Participant 1), transverse colon (Participant 4) and small bowel (Participant 6), correlating with histological GI-GVHD. Response [^18^F]GE-180 PET/CT: Complete metabolic resolution of avidity in sigmoid (Participant 1, clinical complete response); partial metabolic resolution in transverse colon (Participant 4, clinical partial response); similar or partial increase in avidity in duodenum (Participant 6, complete response at time of PET with subsequent recurrence of GI-GVHD). [^18^F]GE-180 PET/CT was performed using a Siemens Biograph mCT Flow 128 Edge 4R PET/CT scanner, and PET SUV window threshold level is set 0-8.

We recently performed a prospective pilot study of PET/CT using the novel radiotracer [^18^F]GE-180 (GE Healthcare, Chicago USA) [65] in adult HCT recipients with suspected acute GI-GVHD [66] (representative images reproduced with permission in Fig. 1). [^18^F]GE-180 targets the translocator protein 18 kDa (tryptophan-rich sensory protein oxygen sensor; TSPO) [67], which is an outer mitochondrial membrane protein that is overexpressed by enterocytes in inflammatory bowel diseases (IBD) [68, 69] as a self-preservation response to tumour necrosis factor (TNF) and interleukin (IL)-8-driven reactive oxygen species (ROS) production and apoptosis [70]. We hypothesised that TSPO expression is increased in enterocytes during acute GI-GVHD and can thus serve as a PET biomarker. Eight participants underwent [^18^F]GE-180 PET/CT for diagnosis and response assessment; images were correlated with histology and clinical findings. We showed that GI tract avidity for TSPO ligand correlated with histology in 75% of participants, with the sensitivity highest in small bowel (86%) and colon (72%). Immunohistochemistry for TSPO showed that enterocytic TSPO expression was significantly increased in GI-GVHD compared to non-GVHD specimens (*p* < 0.0001) in the colon (*p* = 0.0002) and, potentially, the rectum (*p* = 0.06). Our results suggested that, similar to IBD, enterocytic TSPO protein expression is increased in colorectal GI-GVHD; however, differences in SUVmax were modest, rendering PET interpretation challenging.

## Challenges in PET research and implementation in GI-GVHD

Whilst PET evaluation of GI-GVHD is feasible and may be clinically informative, these studies highlight important considerations for future research design and clinical implementation (Table 2). A key challenge is determining the optimal method of quantitatively reporting GI tract uptake in comparison to reference tissue. Radiotracer uptake may vary across GI tract regions in both physiologic and disease states due to differing background expression, tissue vascularity, and partial volume effect (PVE); PVE reflects the effect of volume, in this case bowel wall thickness, on background radiotracer uptake [71]. In both our and Cherk’s studies, quantitative avidity in the GI tract progressively declined from stomach to rectum, with higher rates of false positive and false negative in stomach and rectum respectively compared to the more consistent findings seen in small bowel and colon, illustrating that this issue is likely not just specific to radiotracer selection but an inherent challenge when assessing the heterogeneous GI tract. Consequently, conventional quantitative reporting techniques (such as SUV > 1.5x reference, or SUV above a pre-defined cut-off) may over or under-estimate radiotracer uptake in some GI tract regions, potentially diluting the sensitivity and specificity outcomes of PET studies assessing the entire GI tract.

**Table 2:** Challenges for PET research and implementation in GI-GVHD.

Issue	Description
Molecular target	Ideal target is specific to GI-GVHD biology, present in significantly higher levels in pathological compared to reference and non-GVHD tissues, sustained throughout the course of active GI-GVHD, and readily accessible at a cellular/molecular level.
Radiotracer properties	Properties of the radiotracer compound’s radionuclide (half-life, decay, ligand chemical compatibility) and ligand (non-agonist to target molecule) influence research feasibility and clinical deliverability.
Radiotracer manufacture	Radiotracer availability at short notice is desirable for clinical use. Radiotracers with short half-life generally require on-site manufacture.
Infrastructure	Institutional PET imaging modalities (CT+/− MR) require optimal spatial resolution for assessing the GI tract.
GI tract PET assessment	Radiotracer uptake may vary across GI tract regions in both physiologic and disease states. Specific reference tissues and mathematical quantitative assessment methods for different radiotracers are likely required. Reference tissue should contain a consistently low level of target molecule expression in both GI-GVHD and non-GI-GVHD individuals.
Clinical expertise	Clinical PET interpretation, including positive and negative cut-offs, are dependent on the radiotracer and require institutional expertise.
Effect of treatment	Depending upon molecular target, some anti-GVHD treatments may reduce molecule expression and thus PET radiotracer uptake.
Cost	Radiotracer development and research entail significant time and cost, similar to that of novel pharmaceutical agents. Clinical manufacture costs vary depending upon radiotracer.

Another challenge is that GI-GVHD is an end-organ manifestation of a blood-based disease; radiotracers targeting markers of T-cell activation (for example) in affected GI tract regions are likely to also report increased uptake in haematopoietic tissues such as mediastinal blood pool (MBP), liver, spleen and lymphoid organs. MBP and liver are typical reference tissues for conventional PET radiotracers, but it may be challenging to report a relative increase in GI uptake against a reference tissue that also contains increased uptake due to the same pathophysiology. To be clinically meaningful in individual patients, the candidate radiotracer would demonstrate significantly increased uptake within the pathological GI tract region compared to a non-GVHD reference tissue, and compared to any non-GVHD GI tract regions - including normal GI tract, and those affected by other differential diagnoses such as mucositis or CMV colitis.

With these and other factors in mind, careful consideration should be taken when designing studies of PET in GI-GVHD. Reference tissue ideally should contain a consistent, similarly low level of target molecule expression in both GI-GVHD and non-GI-GVHD states. Co-reporting of both qualitative and quantitative assessments is advisable, for reproducibility and subsequent implementation. Specific mathematical methods of correction may be required to accurately compare some GI tract regions’ uptake with that of reference tissue. PET reporting protocols could be limited to only assess areas of clinical interest (e.g. colon, ileum, and/or endoscopically inaccessible small bowel). Anatomic co-registration using imaging modalities with high spatial resolution, such as MRI instead of CT, may also improve precision and thus reporting accuracy.

## Combining PET with established GI-GVHD biomarkers

GI-GVHD biology is sufficiently complex that there is a surfeit of potential biomarker options for radiotracer development. During the last decade, numerous research groups have identified a multitude of potential aGVHD biomarkers. These include: serum cytokines (REG3α and ST2; the MAGIC Algorithm Probability/Ann Arbor Score [MAP/AA]), where increased levels can predict GVHD onset, and are associated with increased severity, inferior response and survival [72, 73]; and stool assessment of GI tract microbiome, where decreased microbial diversity has been correlated with GI-GVHD incidence, severity, and mortality [74–76]. Insights into the topography of gene and protein expression during GI-GVHD using novel techniques such as spatial transcriptomics [77, 78], may yet yield other tissue-based biomarkers. Whilst there are potential challenges in translating measurement of cytokine or stool biomarkers into routine clinical practice, these represent a very promising advance in GI-GVHD prediction and diagnosis and are poised to be integrated into clinical practice.

Whilst acknowledging their advantages and disadvantages (Table 3), novel diagnostic modalities have great potential to be more informative than conventional techniques. In particular, our rapidly expanding understanding of GI-GVHD biology and identification of potential biomarkers offers an opportunity to develop PET in parallel. Despite the challenges discussed earlier, PET assessment of tissue-based biomarkers has the potential to have distinct advantages over peripheral blood or biopsy sampling: namely, the capacity to evaluate the entire GI tract, define the extent and location of pathological lesions, and return a result in real-time on the day of the PET examination.

**Table 3:** Comparison of diagnostic modalities in GI-GVHD.

Modality	Examples	Advantages	Disadvantages
Endoscopic biopsy	Flexible sigmoidoscopy and histology	Gold standard for histological confirmation; highly specific.	Suboptimal sensitivity due to sampling error (inaccessible sites, patchy involvement); 48–72 h turnaround time for biopsy results; procedural risk; minimal prognostic information.
Conventional Imaging	CT, MR, US	Non-invasive, can evaluate inaccessible sites; sensitive; fast turnaround time for results; minimal patient risk.	Non-specific for GI-GVHD.
Positron Emission Tomography (PET)	[^18^F]FDG [^18^F]GE-180 [^18^F]AraG [^64^Cu]OX40mAb	Non-invasive, can evaluate inaccessible sites; potential for high sensitivity and specificity by radiotracer selection; fast turnaround time for results; likely minimal patient risk, potential for GI-GVHD prediction and/or prognostication.	Cost of radiotracer investigation and/or production; radiotracer availability; requirement for radiotracer-specific quantitative assessment protocols; sensitivity/specificity for individual radiotracers needs evaluation in clinical trials.
Blood- and/or stool- based biomarkers	REG3α, ST2, Faecal microbiome	Established link to GI-GVHD biology; predicts GI-GVHD onset and/or severity.	Diagnostic specificity, definition of “positive” test and optimal timing of measurement yet to be fully defined; turnaround time for individual patient results; requirement for validation in local populations.

## Future directions and conclusions

As new radiotracers are developed, PET’s potential role may evolve beyond non-invasive accurate GI-GVHD diagnosis. Significant GI tract uptake at a clinically meaningful timepoint, with or without contemporaneous blood and/or stool biomarker assessment, may aid prediction of GI-GVHD onset and thus justify trials of early intervention treatment. PET radiotracers with high correlation for specific tissue-based biomarkers may be utilised as surrogate measurements for existing laboratory assays, where PET’s fast turnaround time may be significantly shorter than that of the corresponding laboratory biomarker assay. Combining multiple radiotracers could feasibly assess the relative contribution to symptoms when GVHD and CMV co-exist. For patients treated for GI-GVHD, PET radiotracer uptake may be a more informative assessment of response than crude measurement of daily stool output; for example, by demonstrating initial increase and then subsequent reduction in excess GI tract avidity on serial PET assessment, or by demonstrating excess SUVmax beyond a stipulated threshold at a meaningful timepoint.

More broadly, tissue-based biomarker detection by molecular imaging has the potential to not only define sites of GI-GVHD but also to understand its biology, and may be a highly informative tool to guide further GI-GVHD research. For now, given the limitations of endoscopy for GI-GVHD diagnosis, ongoing research into novel PET radiotracers are warranted, and represent an exciting potential advance in the field.
